# Cutaneous Metaplastic Synovial Cyst: A Case Report 

**Published:** 2017-01-19

**Authors:** Mina Majdi, Hana Saffar, Alireza Ghanadan

**Affiliations:** 1 *Dept. of Pathology, Cancer Institute, Imam Khomeini Hospital Complex, Tehran, Iran*; 2 *Dept. of Dermatopathology, Razi Skin Hospital, Tehran, Iran*

**Keywords:** Metaplastic Synovial Cyst, Immunohistochemistry, Synovial Cyst

## Abstract

Cutaneous metaplastic synovial cyst (CMSC), presents as a solitary, tender subcutaneous nodule that usually occurs at the site of previous surgery or trauma. Histologically, the lesion is characterized by a cystic structure with villous-like projections that lined by metaplastic synovial tissue. The main cause remains unclear, but trauma is presumed to be a precipitating factor, as most reported cases have a history of antecedent cutaneous injury. Here we present a case of CMSC in a 51 year old man, presented with a painless deep-seated dermal nodule in the medial aspect of left ankle without history of any trauma or surgery in this site. Immuno-histochemistry study reveals positive reaction for CD68 in the cystic wall and negative reactions for S-100. CMSC is a unique lesion and worthy to attention, and should be included in the differential diagnosis of deep dermal cutaneous cysts.

## Introduction

Cutaneous metaplastic synovial cyst (CMSC) is an unusual non-neoplastic and benign subcutaneous cystic lesion that usually occurs as posttraumatic solitary and tender nodule or in the site of previous surgery. This entity is different from synovial cyst described as herniation of synovium through joint capsule (1). 

Histologically, CMSC is characterized by cystic space lined by metaplastic synovial tissue accompanied by protrusion of villous structure to cystic spaces and fibrin deposition. The cyst wall lining is composed of alternating foci of cellular area containing polygonal cells with eosinophilic cytoplasm and fibroblast like cells as well as areas without distinct lining only covered by fibrinoid material (1, 2).

Although certain etiology of the disease is not well-known until now, the majority of cases had history of local trauma or surgical manipulation at the same site, therefore mentioned factors proposed as probable etiology of the disease. Therefore, some cases are reported in Ehlers-Danlos syndrome (3), rheumatoid arthritis (4) and in the site of hyaluronic acid filler injection site (5). CMSC is usually treated by surgical excision with rare recurrence rate. 

Here, we reported an unusual case of CMSC with no history of trauma or surgical procedure.

## Case report

**Study population**

A 51 year old man who presented with painless subcutaneous mass like lesion in the medial aspect of left ankle was referred to the Imam Khomeini Hospital Complex, Tehran University of Medical Sciences, Tehran, Iran in 2015. He has no history of significant trauma or any surgical procedure in site of lesion. By physical examination, a solitary well-delineated cystic dermal nodule measuring 3.5×1.5 cm was founded. The overlying skin was normal in physical examination without erythema, warmth or tenderness. The lesion was excised by surgery. Through gross examination, we found out a dermal cyst covered by skin with smooth internal surface containing serosal colorless fluid. 

**Fig. 1:**
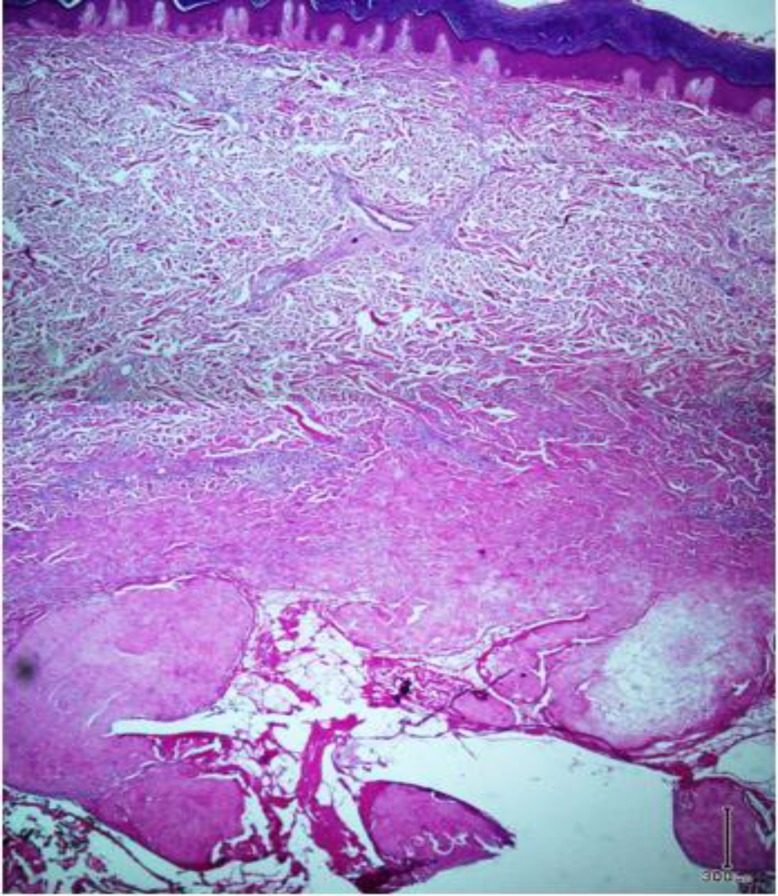
Low power view of specimen biopsy shows deep-seated dermal cyst with villus-like projections into luminal space with surrounding fibrosis and inflammation (Hematoxylin-eosin, original magni-fication × 40

**Fig. 2:**
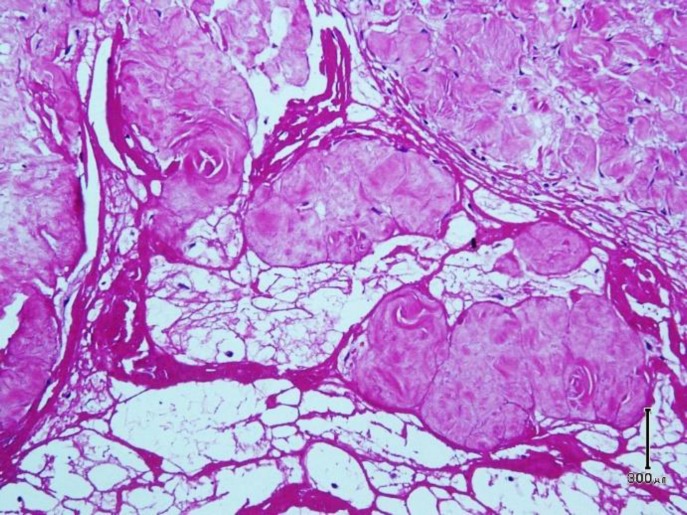
High power of specimen biopsy shows fibrinoid materials (network) among villus-like projections with hypocellular hyalinized stroma (Hematoxylin-eosin, original magnification × 200

**Fig. 3:**
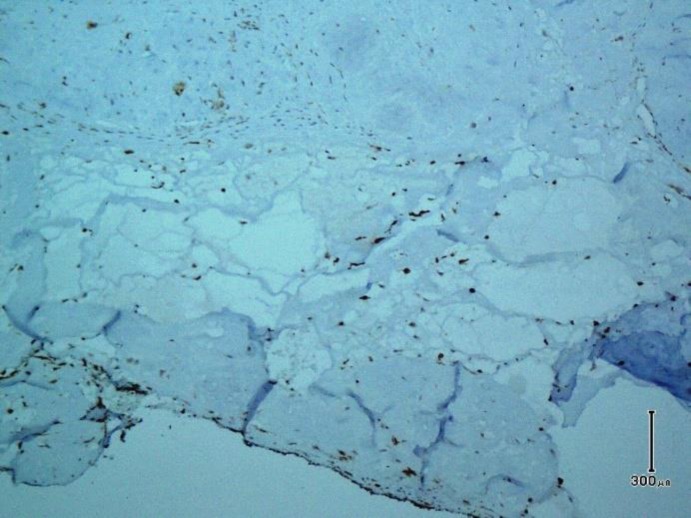
Immunostaining of CD68 in histiocytes (zone 1) of the cyst wall and lining cells (Immunohistochemistry, original magnification × 200

**Fig. 4:**
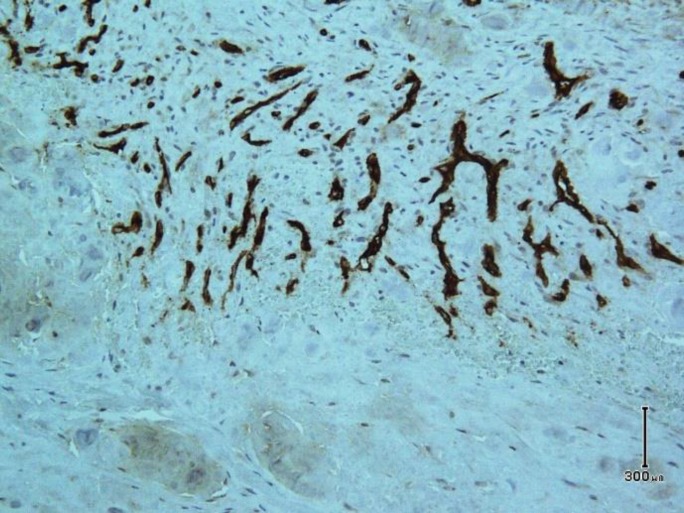
CD34 immunostaining in vascular channels (zone 3) of the cyst wall (Immunohistochemistry, original magnification × 200

Microscopic examination shows skin tissue with underlying deep-seated dermal cyst devoid of true epithelial cells with villus-like projections into cystic space (Fig. 1). The cyst contains fibrinoid materials accompanied by villus-like projections containing histiocytes and mononuclear cells with scattered fibroblasts. Deep dermis surrounding the cyst wall reveals degenerated collagen bundles with fibrosis and inflammation. The stroma was hypocellular and hyalinized in some areas with foci of fibrin deposition (Fig. 2). Immunohistochemistry staining reveals immunoreaction of stromal cells with vimentin antibody and negative reaction with S-100 marker. CD68 marker was positive in histiocytes of cyst wall (Fig. 3) and CD34 marker was positive in vascular channels of the cyst wall (Fig. 4). We diagnosed CMSC based on clinical data, histopathologic examination, and immunohistochemistry findings.

## Discussion

The first case of CMSC was described by Gonzalez et al. in 1987 (1). Since then several studies (Will be discussed later) have reported this rare entity, most of them associated with known predisposing factor. Our case of CMSC was interesting because we found a painless lesion without history of well-known predisposing factor [trauma or previous surgical manipulation]. A case of spontaneous CMSC was reported without history of local trauma (2).

Besides, a child was presented with Ehlers-Danlos syndrome and CMSC without local trauma or previous surgery in site of the lesion. Inappropriate collagen synthesis was offered as probable etiologic factor (3). Overall, 16 patients of CMSC were reviewed in the English literature which most of them had history of pre-existing factor for example surgical scar, burning, foreign body inclusion or repetitive digital manipulation (6). A case of multiple, bilateral CMSC in rheumatoid arthritis was presented as the first report of multiple lesions (7). Although inflammation has been one of important factors of host defense against physical trauma, in such case chronic prolonged inflammatory process might be led to problem in healing process (8).

A case of CMSC in zygomatic region was analyzed with history of hyaluronic acid filler injection. They proposed immune system inflammatory response to tissue damage due to filler injection as cause of this lesion (5). Synovial like metaplasia is also reported in the region of breast implant and silicon prostheses (9). Immunohistochemistry (IHC) technique is used to demonstrate the origin of the lesion triggered by silicon rather than surgical scar (positive for vimentin, CD68, S-100). CMSC can occur around breast implant capsule probably unrelated to the implant. In this study, post-surgical scar site is more etiology probable of the lesion confirmed by IHC technique to reveal immunoreaction for mesenchymal lineage vs. epithelial origin (positive for vimentin and negative for CD34 and CD68). The normal synovial lining is positive for CD68 and negative for S-100 that confirmed the synovial cells of a normal synovium are derived from histiocytes of bone marrow, whereas fibroblasts are from local mesenchymal cells (10, 11). Positive immunoreaction for vimentin and CD68 in cells lining the cyst walls supports the similarities between normal and metaplastic synovium (8). 

Histologically, CMSC is a pseudocyst devoid of true epithelial lining characterized by multiple villous structures resembling synovial membrane. The cyst lining has variable cellularity from hypocellular to hypercellular areas contain a mixture of fibroblasts, mononuclear inflammatory cells, epithelioid cells and multinucleated giant cells. The hypercellular area has zonal pattern from the luminal to the outer zone include polymorphic epithelioid and multinucleated giant cells (zone 1 or luminal), inflammatory cells (zone 2), vascular tissue (zone 3) and fibroconnective tissues (zone 4). The hypocellular areas are characterized by hyalinized zone of connective tissue and fibrin deposition surrounded by subcutaneous tissue with reactive fibrosis (12). Immunohistochemistrically, cyst wall lining exhibits strong positive reaction for vimentin and negative immunoreaction for keratin, S-100 and CEA (7, 11). However, diagnosis of CMSC using immunohistochemistry study is not mandatory and it can be diagnosed using histopathological findings. 

The diagnosis is cumbersome because CMSC is unfamiliar to dermatologist. It should be differentiated from more common lesions with similar clinical manifestation. Differential diagnosis of similar lesions in this region includes synovial cyst of ankle, which can form a mass like lesion under skin. It may or may not be tender depending on its size and location. Histologically synovial cyst is lined by synovial cells (positive for CD68) with communication to the adjacent joint usually involved by osteoarthrosis or inflammatory process like rheumatoid arthritis (12).

The other differential diagnosis is ganglion cyst that is common in the ankle and is described by cystic lesion containing mucinous fluid and discontinuous layer of pseudosynovial cells flattened and surrounded by connective tissue. Because of histologic findings and clinical sign and symptom, bursitis of the ankle can lie in differential diagnosis of painful mass-like lesions of this region. In comparison with CMSC bursitis is located deeper with more symptoms rather than CMSC.

In case of history of surgical intervention, suture granuloma as a granulomatous reaction to foreign materials (starch, talc, suture, tattoo, etc.) can be clinically similar to CMSC or even can be misdiagnosed as recurrence of primary tumor previously excised at the same site (13). We diagnosed our case mainly according to histologic features and immunohistochemistry study that vessels were positive for CD34 and stroma cells show immunoreaction with CD68. 

## Conclusion

The diagnosis of CMSC in any dermal or subcutaneous mass-like or cystic lesion in periarticular area should be based on combination of clinical history, careful physical examination, and histologic findings as well as exclusion of clinically and histologically similar lesions of this region. 

## Conflict of Interests

The authors declare that there is no Conflict of Interests. 
